# *P*,*P*-Bis[4-(dimethyl­amino)­phen­yl]-*N*,*N*-bis­(propan-2-yl)phosphinic amide

**DOI:** 10.1107/S1600536812050398

**Published:** 2013-01-09

**Authors:** Stephen J. Evans, C. Alicia Renison, D. Bradley G. Williams, Alfred Muller

**Affiliations:** a Research Centre for Synthesis and Catalysis, Department of Chemistry, University of Johannesburg (APK Campus), PO Box 524, Auckland Park, Johannesburg, 2006, South Africa

## Abstract

The mol­ecular structure of the title compound, C_22_H_34_N_3_OP, adopts a distorted tetra­hedral geometry at the P atom, with the most noticeable distortion being for the O—P—N angle [117.53 (10)°]. An effective cone angle of 187° was calculated for the compound. In the crystal, weak C—H⋯O inter­actions create infinite chains along [100], whereas C—H⋯π inter­actions propagating in [001] generate a herringbone motif.

## Related literature

For the synthesis of ligands derived from phosphinic amides, see: Williams *et al.* (2009 ▶). For background to DoM technology, see: Snieckus (1990 ▶). For cone angles, see: Tolman (1977 ▶); Otto (2001 ▶).
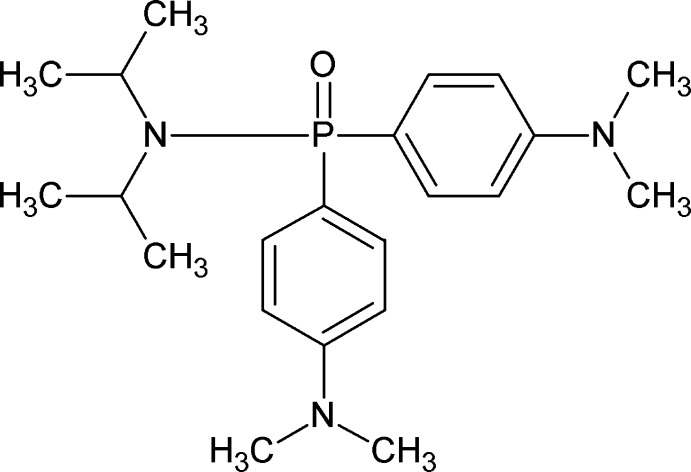


## Experimental

### 

#### Crystal data

C_22_H_34_N_3_OP
*M* *_r_* = 387.49
Orthorhombic, 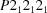

*a* = 6.2960 (4) Å
*b* = 16.6389 (8) Å
*c* = 19.9475 (11) Å
*V* = 2089.7 (2) Å^3^
*Z* = 4
Mo *K*α radiation
μ = 0.15 mm^−1^
*T* = 100 K
0.13 × 0.11 × 0.1 mm

#### Data collection

Bruker X8 APEXII 4K KappaCCD diffractometer
Absorption correction: multi-scan (*SADABS*; Bruker, 2004 ▶) *T*_min_ = 0.981, *T*_max_ = 0.985
18896 measured reflections
5212 independent reflections
3840 reflections with *I* > 2σ(*I*)
*R*_int_ = 0.074

#### Refinement

*R*[*F* ^2^ > 2σ(*F* ^2^)] = 0.050
*wR*(*F* ^2^) = 0.110
*S* = 1.04
5212 reflections
252 parameters
H-atom parameters constrained
Δρ_max_ = 0.30 e Å^−3^
Δρ_min_ = −0.35 e Å^−3^
Absolute structure: Flack (1983 ▶), 2224 Friedel pairs
Flack parameter: 0.11 (10)

### 

Data collection: *APEX2* (Bruker, 2005 ▶); cell refinement: *SAINT-Plus* (Bruker, 2004 ▶); data reduction: *SAINT-Plus* and *XPREP* (Bruker, 2004 ▶); program(s) used to solve structure: *SIR97* (Altomare *et al.*, 1999 ▶); program(s) used to refine structure: *SHELXL97* (Sheldrick, 2008 ▶); molecular graphics: *DIAMOND* (Brandenburg & Putz, 2005 ▶); software used to prepare material for publication: *publCIF* (Westrip, 2010 ▶) and *WinGX* (Farrugia, 2012 ▶).

## Supplementary Material

Crystal structure: contains datablock(s) global, I. DOI: 10.1107/S1600536812050398/bt6869sup1.cif

Structure factors: contains datablock(s) I. DOI: 10.1107/S1600536812050398/bt6869Isup2.hkl

Supplementary material file. DOI: 10.1107/S1600536812050398/bt6869Isup3.cml

Additional supplementary materials:  crystallographic information; 3D view; checkCIF report

## Figures and Tables

**Table 1:** Hydrogen-bond geometry (Å, °) *Cg*1 and *Cg*2 are the centroids of the C11—C16 and C21—C26 rings, respectively.

*D*—H⋯*A*	*D*—H	H⋯*A*	*D*⋯*A*	*D*—H⋯*A*
C12—H12⋯O1^i^	0.95	2.59	3.493 (3)	159
C33—H33*A*⋯O1^i^	0.98	2.58	3.501 (3)	158
C18—H18*A*⋯*Cg*1^ii^	0.98	2.96	3.821 (2)	148
C18—H18*C*⋯*Cg*2^ii^	0.98	2.97	3.915 (3)	162
C27—H27*C*⋯*Cg*1^iii^	0.98	2.69	3.468 (3)	137

Symmetry codes: (i) 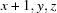; (ii) 
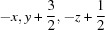
; (iii) 
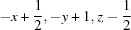
.

**Fig. 1.**
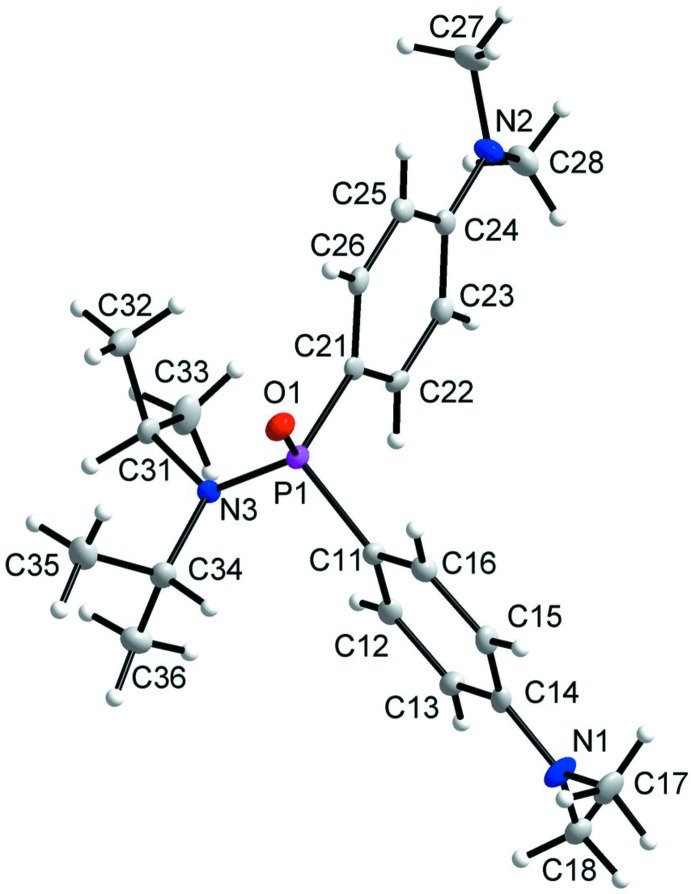
A view of the title complex, showing the atom-numbering scheme and 50% probability displacement ellipsoids.

**Fig. 2.**
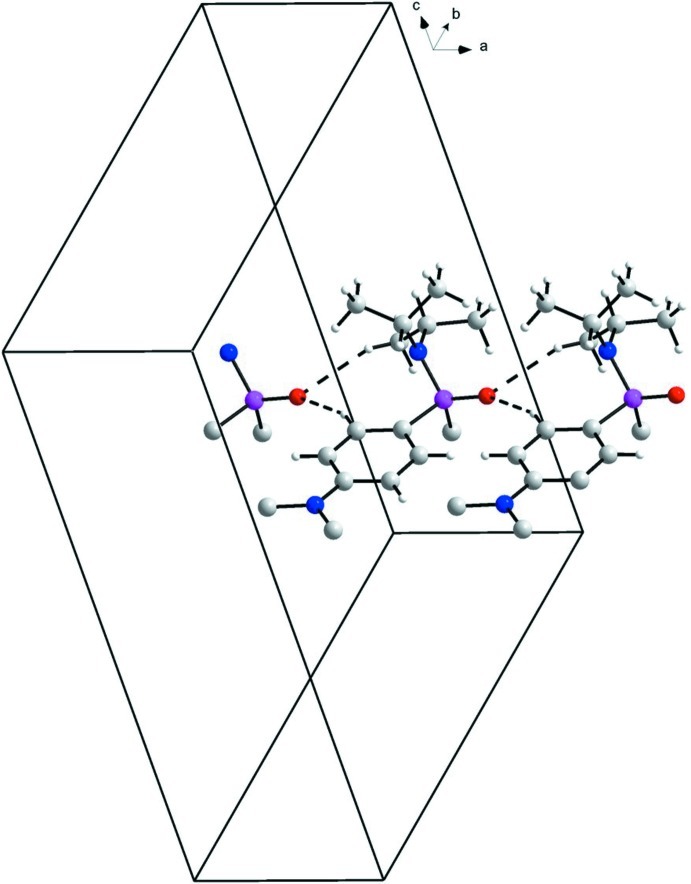
Packing diagram showing only the C—H···O interactions (indicated by dashed lines) creating infinitely long chains along the [100] direction.

**Fig. 3.**
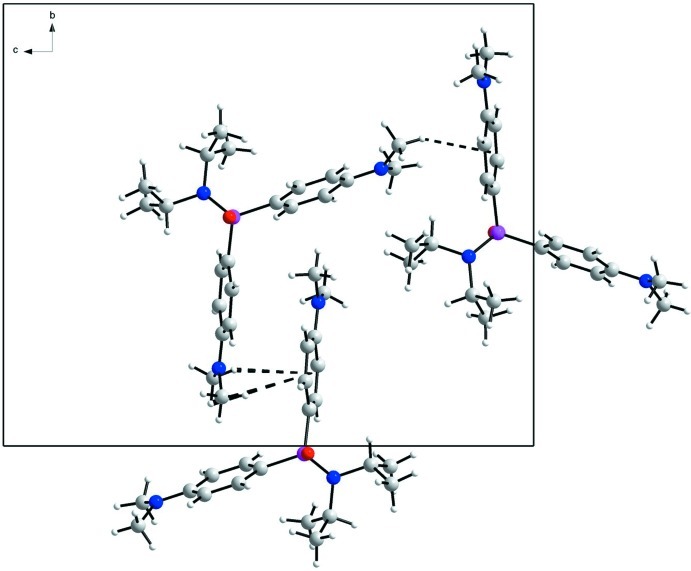
Packing diagram showing only the C—H···π interactions (indicated by dashed lines) propagating along the [001] direction.

**Fig. 4.**
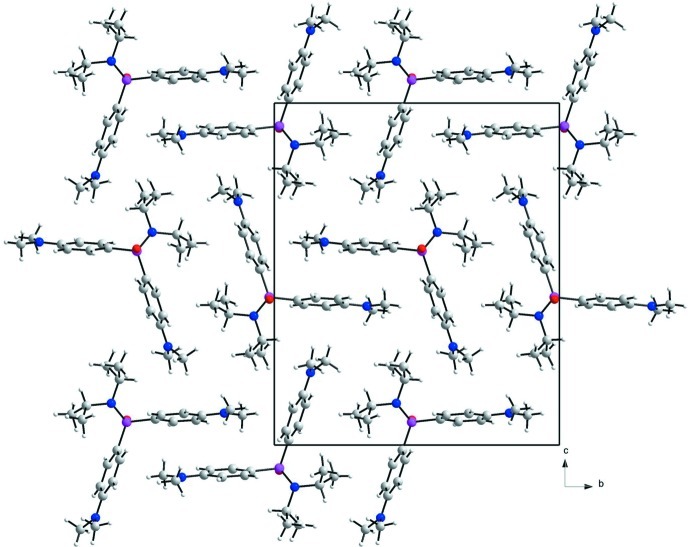
Packing diagram showing the generated herring-bone motif from the interactions.

## supplementary crystallographic information

### Comment

An expedient rapid synthesis of ligands derived from phosphinic amides that were found to be suitable for the Suzuki-Miyaura reactions at low palladium catalyst loadings was developed (Williams *et al.*, 2009). The brief practical synthesis affords arylphosphine ligands resistant to oxidation and hydrolysis while maintaining high catalyst activity. The synthesis rests strongly on DoM technology (Snieckus, 1990) making use of a directing group that is highly underrepresented in this type of chemistry. We envisioned that the use of phosphinic amides as directing groups, together with phosphinous chloride (Cy_2_PCl) electrophiles would allow the synthesis of sterically hindered phosphines that are stable to hydrolysis and oxidation. Manipulating the phosphinic amide functionality has been shown to influence the catalytic performance of the resulting alkyl phosphine ligands and the structure reported here is one of the substrates for further ligand studies.

The title compound (see Fig. 1) crystallizes in the orthorhombic space group *P*2_1_2_1_2_1_ (*Z*=4) with its molecules adopting a distorted tetrahedral arrangement about the phosphorus atom. The O3—P1—N3 angle of 117.53 (10)° shows this distorted arrangement the most prominent, and it is further exemplified by the twisted orientation of the bulky amide substituent to fit into the coordination sphere of the phosphorus atom (seen from the torsion angles C34—N3—P1—O1 = -63.71 (19)° and C31—N3—P1—O1 = 87.2 (2)° respectively). The most common method used for determining the steric behaviour of a phosphane ligand is the Tolman cone angle (Tolman, 1977). We used the geometry from the title compound and adjusted the P═O distance to 2.28 Å (the average Ni—P distance used in the original Tolman model) to cancel the bias this may have on the calculated cone angle value. In this way we obtain the effective cone angle (Otto, 2001) value of 187°. Several weak C—H···O interactions are observed in the crystal lattice creating infinitely long chains along the [100] direction (Fig. 2). Additional C—H···π interactions are also observed which propagates along the [001] direction in the crystal lattice (Fig. 3). These interactions (summarized in Table 1) generate a herring-bone packing motif (Fig. 4).

### Experimental

Diisopropyl amine (1.55 ml, 5.53 mmol) was added to a solution of PCl_3_ (241 µ*L*, 2.77 mmol) in toluene (250 ml) at 0 °C. The mixture was allowed to stir for 2 h at room temperature. In a separate flask *p*-bromo-*N,N*-dimethylaniline (1.728 g, 8.63 mmol) in THF (5 ml) was added to magnesium turnings (200 mg, 8.22 mmol) in THF (5 ml) and heated to 65 °C. The reaction was initiated with a crystal of iodine and the suspension allowed to stir for 3 h at that temperature. Once the magnesium had fully reacted the two solutions were combined and the salts were removed by filtration through a pad of celite under argon.

The solution was cooled to 0 °C and hydrogen peroxide (30%, 15 ml) was added over 20 minutes. The mixture was allowed to stir for a further 1 h. The product was extracted with EtOAc and H_2_O and the solvent removed *in vacuo*. The product was isolated by flash column chromatography (EtOAc).

Crystals were grown by dissolving in a minimal amount of DCM and layering an excess of hexane on top and allowing to stand in a refrigerator until the crystals were formed.

Yield: 60% (yellow solid). ^1^H NMR: (300 MHz, CDCl_3_) δH 7.58 (t, 4H, H2, H2`, H6 and H6`, *J* = 9.9 Hz), 6.61 (d, 4H, H3, H3`, H5 and H5`, *J* = 7.2 Hz), 3.41 (sept, 2H, NCH(CH_3_)_2_, *J* = 6.9 Hz), 2.90 (s, 12H, NCH(CH_3_)_2_), 1.12 (d, 12H, NCH(CH_3_)_2_, *J* = 6.9 Hz). ^13^C NMR: (75 MHz, CDCl_3_) δC 151.6 (d, 2 C, C4 and C4`, *J* = 2.3 Hz), 133.4 (d, 4 C, C2, C2`, C6 and C6`, *J* = 10.6 Hz), 120.3 (d, 2 C, C1 and C1`, *J* = 135.3 Hz), 110.7 (d, 4 C, C3, C3`, C5 and C5`, *J* = 13.0 Hz), 46.5 (d, 2 C. NCH(CH_3_), *J* = 4.3 Hz), 398 (s, 4 C, NCH(CH_3_)_2_, 32.1 (d, 4 C, NCH(CH_3_)_2_, *J* = 2.6 Hz). ^31^P NMR: (121 MHz, CDCl_3_) δP 31.1(S,1P). ElMS: *m/z* 387 [(*M*), 10%], 344 [(M—C_3_H_7_), 12%], 287 [(M—C_6_H_14_N), 100%]. IR: ν (CHCl_3_) 2980, 1262, 1172. HRMS: Calculated: 387.2440 C_22_H_34_N_3_OP Obtained: 387.2445

### Refinement

The aromatic, methine and methyl atoms were placed in geometrically idealized positions (C—H = 0.95–1.0 Å) and constrained to ride on their parent atoms with *U*_iso_(H) = 1.2*U*_eq_(C) for the aromatic and methine H and *U*_iso_(H) = 1.5*U*_eq_(C) for the methyl H respectively. The Flack parameter refined to 0.11 (10).

### Crystal data

**Table:**

C_22_H_34_N_3_OP	*F*(000) = 840
*M**_r_* = 387.49	*D*_x_ = 1.232 Mg m^−^^3^
Orthorhombic, *P*2_1_2_1_2_1_	Mo *K*α radiation, λ = 0.71073 Å
Hall symbol: P 2ac 2ab	Cell parameters from 3113 reflections
*a* = 6.2960 (4) Å	θ = 2.4–25.9°
*b* = 16.6389 (8) Å	µ = 0.15 mm^−^^1^
*c* = 19.9475 (11) Å	*T* = 100 K
*V* = 2089.7 (2) Å^3^	Prism, colourless
*Z* = 4	0.13 × 0.11 × 0.1 mm

### Data collection

**Table:**

Bruker X8 APEXII 4K KappaCCD diffractometer	3840 reflections with *I* > 2σ(*I*)
Graphite monochromator	*R*_int_ = 0.074
φ and ω scans	θ_max_ = 28.3°, θ_min_ = 1.6°
Absorption correction: multi-scan (*SADABS*; Bruker, 2004)	*h* = −4→8
*T*_min_ = 0.981, *T*_max_ = 0.985	*k* = −19→22
18896 measured reflections	*l* = −26→26
5212 independent reflections	

### Refinement

**Table:**

Refinement on *F*^2^	Secondary atom site location: difference Fourier map
Least-squares matrix: full	Hydrogen site location: inferred from neighbouring sites
*R*[*F*^2^ > 2σ(*F*^2^)] = 0.050	H-atom parameters constrained
*wR*(*F*^2^) = 0.110	*w* = 1/[σ^2^(*F*_o_^2^) + (0.0467*P*)^2^] where *P* = (*F*_o_^2^ + 2*F*_c_^2^)/3
*S* = 1.04	(Δ/σ)_max_ = 0.002
5212 reflections	Δρ_max_ = 0.30 e Å^−^^3^
252 parameters	Δρ_min_ = −0.35 e Å^−^^3^
0 restraints	Absolute structure: Flack (1983), 2224 Friedel pairs
Primary atom site location: structure-invariant direct methods	Flack parameter: 0.11 (10)

### Special details

**Table:**

Experimental. The intensity data was collected on a Bruker X8 APEXII 4 K KappaCCD diffractometer using an exposure time of 20 s/frame. A total of 1010 frames were collected with a frame width of 0.5° covering up to θ = 28.33° with 99.9% completeness accomplished.
Geometry. All e.s.d.'s (except the e.s.d. in the dihedral angle between two l.s. planes) are estimated using the full covariance matrix. The cell e.s.d.'s are taken into account individually in the estimation of e.s.d.'s in distances, angles and torsion angles; correlations between e.s.d.'s in cell parameters are only used when they are defined by crystal symmetry. An approximate (isotropic) treatment of cell e.s.d.'s is used for estimating e.s.d.'s involving l.s. planes.
Refinement. Refinement of *F*^2^ against ALL reflections. The weighted *R*-factor *wR* and goodness of fit *S* are based on *F*^2^, conventional *R*-factors *R* are based on *F*, with *F* set to zero for negative *F*^2^. The threshold expression of *F*^2^ > σ(*F*^2^) is used only for calculating *R*-factors(gt) *etc*. and is not relevant to the choice of reflections for refinement. *R*-factors based on *F*^2^ are statistically about twice as large as those based on *F*, and *R*- factors based on ALL data will be even larger.

### Fractional atomic coordinates and isotropic or equivalent isotropic displacement parameters (Å^2^)

**Table:**

	*x*	*y*	*z*	*U*_iso_*/*U*_eq_	
C11	0.7556 (4)	−0.08285 (14)	0.92323 (10)	0.0145 (5)	
C12	0.9663 (4)	−0.10305 (14)	0.91217 (10)	0.0161 (5)	
H12	1.0688	−0.0615	0.9079	0.019*	
C13	1.0314 (4)	−0.18282 (14)	0.90711 (11)	0.0161 (5)	
H13	1.1769	−0.1948	0.8994	0.019*	
C14	0.8830 (4)	−0.24597 (13)	0.91337 (10)	0.0162 (5)	
C15	0.6722 (4)	−0.22578 (13)	0.92635 (11)	0.0166 (5)	
H15	0.5697	−0.2671	0.9322	0.02*	
C16	0.6103 (4)	−0.14573 (14)	0.93076 (11)	0.0164 (5)	
H16	0.4654	−0.1334	0.9391	0.02*	
C17	0.7881 (4)	−0.38862 (14)	0.91197 (13)	0.0247 (6)	
H17A	0.6801	−0.382	0.877	0.037*	
H17B	0.7206	−0.386	0.9562	0.037*	
H17C	0.858	−0.4409	0.9065	0.037*	
C18	1.1650 (5)	−0.34575 (14)	0.89615 (11)	0.0208 (6)	
H18A	1.2488	−0.328	0.9348	0.031*	
H18B	1.218	−0.3195	0.8555	0.031*	
H18C	1.1775	−0.4042	0.8913	0.031*	
C21	0.7295 (4)	0.05075 (13)	1.01511 (11)	0.0145 (5)	
C22	0.9324 (4)	0.04153 (13)	1.04203 (11)	0.0152 (5)	
H22	1.0411	0.0179	1.0155	0.018*	
C23	0.9787 (4)	0.06615 (14)	1.10679 (10)	0.0177 (5)	
H23	1.1183	0.059	1.1239	0.021*	
C24	0.8226 (4)	0.10145 (13)	1.14755 (11)	0.0171 (5)	
C25	0.6184 (4)	0.10968 (14)	1.12077 (11)	0.0181 (6)	
H25	0.5091	0.1328	1.1474	0.022*	
C26	0.5730 (4)	0.08471 (13)	1.05623 (11)	0.0166 (5)	
H26	0.4328	0.0907	1.0394	0.02*	
C27	0.7173 (5)	0.17979 (17)	1.24584 (12)	0.0276 (7)	
H27A	0.5815	0.1519	1.2517	0.041*	
H27B	0.6955	0.2282	1.2187	0.041*	
H27C	0.7739	0.1949	1.2898	0.041*	
C28	1.0833 (4)	0.12767 (16)	1.23701 (12)	0.0253 (6)	
H28A	1.163	0.1708	1.2149	0.038*	
H28B	1.1508	0.0759	1.2273	0.038*	
H28C	1.0826	0.1368	1.2855	0.038*	
C31	0.8422 (4)	0.15896 (13)	0.88452 (11)	0.0178 (5)	
H31	0.8743	0.1784	0.8382	0.021*	
C32	0.6577 (5)	0.21017 (14)	0.90928 (13)	0.0262 (6)	
H32A	0.6189	0.1936	0.9548	0.039*	
H32B	0.5355	0.203	0.8794	0.039*	
H32C	0.6999	0.2669	0.9095	0.039*	
C33	1.0435 (5)	0.17255 (15)	0.92493 (13)	0.0276 (6)	
H33A	1.151	0.133	0.9117	0.041*	
H33B	1.0121	0.1665	0.9728	0.041*	
H33C	1.0973	0.2269	0.9164	0.041*	
C34	0.7688 (4)	0.04290 (14)	0.80697 (11)	0.0187 (6)	
H34	0.7268	−0.015	0.8093	0.022*	
C35	0.5943 (4)	0.08600 (16)	0.76836 (12)	0.0248 (6)	
H35A	0.6317	0.1428	0.7633	0.037*	
H35B	0.46	0.0815	0.7929	0.037*	
H35C	0.5786	0.0614	0.724	0.037*	
C36	0.9802 (4)	0.04601 (15)	0.77057 (12)	0.0244 (6)	
H36A	1.0882	0.0181	0.7972	0.037*	
H36B	1.0225	0.1022	0.7642	0.037*	
H36C	0.9663	0.0198	0.7268	0.037*	
N1	0.9457 (4)	−0.32461 (12)	0.90623 (10)	0.0226 (5)	
N2	0.8668 (4)	0.12702 (12)	1.21223 (9)	0.0207 (5)	
N3	0.7898 (3)	0.07164 (11)	0.87759 (9)	0.0155 (5)	
O1	0.4187 (3)	0.01727 (9)	0.92616 (7)	0.0194 (4)	
P1	0.65325 (10)	0.01773 (4)	0.93260 (3)	0.01474 (14)	

### Atomic displacement parameters (Å^2^)

**Table:**

	*U*^11^	*U*^22^	*U*^33^	*U*^12^	*U*^13^	*U*^23^
C11	0.0215 (13)	0.0097 (11)	0.0122 (11)	−0.0013 (9)	0.0018 (10)	−0.0008 (9)
C12	0.0240 (14)	0.0117 (12)	0.0127 (10)	−0.0043 (11)	−0.0032 (10)	−0.0016 (9)
C13	0.0190 (14)	0.0140 (12)	0.0154 (10)	0.0026 (10)	−0.0004 (10)	−0.0007 (9)
C14	0.0266 (16)	0.0102 (11)	0.0116 (9)	−0.0017 (10)	−0.0041 (10)	0.0009 (8)
C15	0.0230 (14)	0.0112 (11)	0.0155 (11)	−0.0054 (11)	−0.0008 (11)	0.0017 (9)
C16	0.0186 (14)	0.0163 (12)	0.0142 (10)	−0.0017 (10)	0.0001 (11)	−0.0004 (9)
C17	0.0333 (17)	0.0087 (12)	0.0322 (14)	−0.0014 (11)	−0.0030 (12)	−0.0009 (11)
C18	0.0296 (16)	0.0118 (12)	0.0209 (12)	0.0017 (12)	−0.0001 (12)	−0.0014 (9)
C21	0.0254 (15)	0.0054 (12)	0.0126 (10)	−0.0044 (10)	0.0026 (10)	0.0009 (8)
C22	0.0206 (14)	0.0071 (11)	0.0179 (11)	0.0009 (10)	0.0039 (10)	−0.0003 (8)
C23	0.0223 (15)	0.0129 (12)	0.0179 (11)	0.0005 (11)	−0.0009 (10)	0.0030 (9)
C24	0.0287 (16)	0.0089 (11)	0.0136 (10)	−0.0022 (11)	0.0018 (10)	0.0017 (8)
C25	0.0249 (16)	0.0132 (12)	0.0163 (11)	0.0000 (11)	0.0057 (11)	0.0028 (9)
C26	0.0208 (14)	0.0109 (12)	0.0180 (11)	0.0002 (10)	0.0003 (10)	0.0043 (9)
C27	0.0339 (19)	0.0338 (16)	0.0152 (12)	0.0073 (13)	−0.0013 (11)	−0.0078 (11)
C28	0.0310 (18)	0.0296 (16)	0.0153 (11)	0.0006 (12)	−0.0012 (11)	−0.0011 (10)
C31	0.0291 (15)	0.0079 (11)	0.0165 (10)	−0.0023 (11)	0.0022 (11)	0.0011 (8)
C32	0.0367 (17)	0.0144 (13)	0.0275 (12)	0.0034 (13)	0.0108 (13)	0.0031 (10)
C33	0.0352 (17)	0.0160 (13)	0.0317 (14)	−0.0079 (12)	−0.0089 (14)	0.0037 (11)
C34	0.0268 (15)	0.0137 (13)	0.0157 (11)	−0.0002 (11)	−0.0012 (11)	−0.0018 (9)
C35	0.0324 (17)	0.0232 (15)	0.0187 (12)	−0.0005 (12)	−0.0038 (11)	0.0011 (10)
C36	0.0325 (17)	0.0218 (14)	0.0189 (12)	−0.0001 (12)	0.0047 (12)	−0.0028 (10)
N1	0.0260 (13)	0.0086 (10)	0.0331 (11)	−0.0005 (9)	−0.0009 (10)	−0.0024 (9)
N2	0.0266 (13)	0.0231 (12)	0.0124 (9)	0.0042 (10)	0.0003 (9)	−0.0037 (8)
N3	0.0267 (13)	0.0081 (10)	0.0118 (9)	−0.0036 (9)	0.0004 (8)	−0.0021 (8)
O1	0.0211 (10)	0.0136 (8)	0.0235 (8)	0.0005 (7)	−0.0008 (7)	−0.0014 (7)
P1	0.0209 (3)	0.0094 (3)	0.0140 (3)	0.0000 (3)	−0.0002 (3)	−0.0005 (2)

### Geometric parameters (Å, º)

**Table:**

C11—C12	1.386 (3)	C26—H26	0.95
C11—C16	1.398 (3)	C27—N2	1.451 (3)
C11—P1	1.803 (2)	C27—H27A	0.98
C12—C13	1.393 (3)	C27—H27B	0.98
C12—H12	0.95	C27—H27C	0.98
C13—C14	1.412 (3)	C28—N2	1.450 (3)
C13—H13	0.95	C28—H28A	0.98
C14—N1	1.374 (3)	C28—H28B	0.98
C14—C15	1.393 (3)	C28—H28C	0.98
C15—C16	1.391 (3)	C31—N3	1.496 (3)
C15—H15	0.95	C31—C33	1.519 (4)
C16—H16	0.95	C31—C32	1.523 (4)
C17—N1	1.460 (3)	C31—H31	1
C17—H17A	0.98	C32—H32A	0.98
C17—H17B	0.98	C32—H32B	0.98
C17—H17C	0.98	C32—H32C	0.98
C18—N1	1.439 (3)	C33—H33A	0.98
C18—H18A	0.98	C33—H33B	0.98
C18—H18B	0.98	C33—H33C	0.98
C18—H18C	0.98	C34—N3	1.493 (3)
C21—C22	1.395 (3)	C34—C36	1.517 (4)
C21—C26	1.401 (3)	C34—C35	1.521 (3)
C21—P1	1.800 (2)	C34—H34	1
C22—C23	1.386 (3)	C35—H35A	0.98
C22—H22	0.95	C35—H35B	0.98
C23—C24	1.404 (3)	C35—H35C	0.98
C23—H23	0.95	C36—H36A	0.98
C24—N2	1.387 (3)	C36—H36B	0.98
C24—C25	1.399 (3)	C36—H36C	0.98
C25—C26	1.383 (3)	N3—P1	1.658 (2)
C25—H25	0.95	O1—P1	1.4825 (17)
			
C12—C11—C16	117.5 (2)	H28A—C28—H28B	109.5
C12—C11—P1	125.71 (18)	N2—C28—H28C	109.5
C16—C11—P1	116.71 (19)	H28A—C28—H28C	109.5
C11—C12—C13	121.6 (2)	H28B—C28—H28C	109.5
C11—C12—H12	119.2	N3—C31—C33	112.16 (19)
C13—C12—H12	119.2	N3—C31—C32	113.9 (2)
C12—C13—C14	120.5 (2)	C33—C31—C32	112.4 (2)
C12—C13—H13	119.7	N3—C31—H31	105.9
C14—C13—H13	119.7	C33—C31—H31	105.9
N1—C14—C15	121.5 (2)	C32—C31—H31	105.9
N1—C14—C13	120.6 (2)	C31—C32—H32A	109.5
C15—C14—C13	117.9 (2)	C31—C32—H32B	109.5
C16—C15—C14	120.6 (2)	H32A—C32—H32B	109.5
C16—C15—H15	119.7	C31—C32—H32C	109.5
C14—C15—H15	119.7	H32A—C32—H32C	109.5
C15—C16—C11	121.8 (2)	H32B—C32—H32C	109.5
C15—C16—H16	119.1	C31—C33—H33A	109.5
C11—C16—H16	119.1	C31—C33—H33B	109.5
N1—C17—H17A	109.5	H33A—C33—H33B	109.5
N1—C17—H17B	109.5	C31—C33—H33C	109.5
H17A—C17—H17B	109.5	H33A—C33—H33C	109.5
N1—C17—H17C	109.5	H33B—C33—H33C	109.5
H17A—C17—H17C	109.5	N3—C34—C36	111.3 (2)
H17B—C17—H17C	109.5	N3—C34—C35	113.0 (2)
N1—C18—H18A	109.5	C36—C34—C35	112.0 (2)
N1—C18—H18B	109.5	N3—C34—H34	106.7
H18A—C18—H18B	109.5	C36—C34—H34	106.7
N1—C18—H18C	109.5	C35—C34—H34	106.7
H18A—C18—H18C	109.5	C34—C35—H35A	109.5
H18B—C18—H18C	109.5	C34—C35—H35B	109.5
C22—C21—C26	117.6 (2)	H35A—C35—H35B	109.5
C22—C21—P1	124.24 (18)	C34—C35—H35C	109.5
C26—C21—P1	118.10 (19)	H35A—C35—H35C	109.5
C23—C22—C21	121.2 (2)	H35B—C35—H35C	109.5
C23—C22—H22	119.4	C34—C36—H36A	109.5
C21—C22—H22	119.4	C34—C36—H36B	109.5
C22—C23—C24	121.1 (2)	H36A—C36—H36B	109.5
C22—C23—H23	119.5	C34—C36—H36C	109.5
C24—C23—H23	119.5	H36A—C36—H36C	109.5
N2—C24—C25	120.6 (2)	H36B—C36—H36C	109.5
N2—C24—C23	121.8 (2)	C14—N1—C18	121.5 (2)
C25—C24—C23	117.6 (2)	C14—N1—C17	119.4 (2)
C26—C25—C24	121.0 (2)	C18—N1—C17	119.0 (2)
C26—C25—H25	119.5	C24—N2—C28	120.5 (2)
C24—C25—H25	119.5	C24—N2—C27	119.1 (2)
C25—C26—C21	121.4 (2)	C28—N2—C27	116.6 (2)
C25—C26—H26	119.3	C34—N3—C31	114.68 (17)
C21—C26—H26	119.3	C34—N3—P1	113.89 (16)
N2—C27—H27A	109.5	C31—N3—P1	125.34 (15)
N2—C27—H27B	109.5	O1—P1—N3	117.53 (10)
H27A—C27—H27B	109.5	O1—P1—C21	110.27 (11)
N2—C27—H27C	109.5	N3—P1—C21	107.57 (11)
H27A—C27—H27C	109.5	O1—P1—C11	110.03 (11)
H27B—C27—H27C	109.5	N3—P1—C11	104.36 (11)
N2—C28—H28A	109.5	C21—P1—C11	106.42 (11)
N2—C28—H28B	109.5		
			
C16—C11—C12—C13	1.5 (3)	C23—C24—N2—C27	165.3 (2)
P1—C11—C12—C13	178.49 (16)	C36—C34—N3—C31	66.8 (3)
C11—C12—C13—C14	−0.2 (3)	C35—C34—N3—C31	−60.2 (3)
C12—C13—C14—N1	177.9 (2)	C36—C34—N3—P1	−139.05 (18)
C12—C13—C14—C15	−1.6 (3)	C35—C34—N3—P1	93.9 (2)
N1—C14—C15—C16	−177.4 (2)	C33—C31—N3—C34	−123.5 (2)
C13—C14—C15—C16	2.0 (3)	C32—C31—N3—C34	107.4 (2)
C14—C15—C16—C11	−0.8 (3)	C33—C31—N3—P1	85.7 (3)
C12—C11—C16—C15	−1.0 (3)	C32—C31—N3—P1	−43.4 (3)
P1—C11—C16—C15	−178.30 (17)	C34—N3—P1—O1	−63.71 (19)
C26—C21—C22—C23	−0.9 (3)	C31—N3—P1—O1	87.2 (2)
P1—C21—C22—C23	−177.98 (17)	C34—N3—P1—C21	171.21 (16)
C21—C22—C23—C24	−0.2 (3)	C31—N3—P1—C21	−37.9 (2)
C22—C23—C24—N2	−179.4 (2)	C34—N3—P1—C11	58.44 (18)
C22—C23—C24—C25	1.0 (3)	C31—N3—P1—C11	−150.6 (2)
N2—C24—C25—C26	179.7 (2)	C22—C21—P1—O1	165.50 (18)
C23—C24—C25—C26	−0.7 (3)	C26—C21—P1—O1	−11.5 (2)
C24—C25—C26—C21	−0.4 (3)	C22—C21—P1—N3	−65.2 (2)
C22—C21—C26—C25	1.2 (3)	C26—C21—P1—N3	117.78 (18)
P1—C21—C26—C25	178.44 (17)	C22—C21—P1—C11	46.2 (2)
C15—C14—N1—C18	−176.9 (2)	C26—C21—P1—C11	−130.84 (18)
C13—C14—N1—C18	3.7 (3)	C12—C11—P1—O1	165.47 (17)
C15—C14—N1—C17	0.3 (3)	C16—C11—P1—O1	−17.5 (2)
C13—C14—N1—C17	−179.1 (2)	C12—C11—P1—N3	38.5 (2)
C25—C24—N2—C28	−172.3 (2)	C16—C11—P1—N3	−144.44 (17)
C23—C24—N2—C28	8.1 (3)	C12—C11—P1—C21	−75.1 (2)
C25—C24—N2—C27	−15.2 (3)	C16—C11—P1—C21	101.96 (19)

### Hydrogen-bond geometry (Å, º)

*Cg*1 and *Cg*2 are the centroids of the C11—C16 and C21—C26 rings, respectively.

**Table:**

*D*—H···*A*	*D*—H	H···*A*	*D*···*A*	*D*—H···*A*
C12—H12···O1^i^	0.95	2.59	3.493 (3)	159
C33—H33*A*···O1^i^	0.98	2.58	3.501 (3)	158
C18—H18*A*···*Cg*1^ii^	0.98	2.96	3.821 (2)	148
C18—H18*C*···*Cg*2^ii^	0.98	2.97	3.915 (3)	162
C27—H27*C*···*Cg*1^iii^	0.98	2.69	3.468 (3)	137

Symmetry codes: (i) *x*+1, *y*, *z*; (ii) −*x*, *y*+3/2, −*z*+1/2; (iii) −*x*+1/2, −*y*+1, *z*−1/2.
